# Data relating to emissions of polychlorinated dibenzo-*p*-dioxins (PCDDs) and polychlorinated dibenzofurans (PCDFs) from industrial boilers

**DOI:** 10.1016/j.dib.2018.12.021

**Published:** 2018-12-13

**Authors:** Siwatt Pongpiachan, Teeraporn Wiriwutikorn, Phonethip Phetsomphou, Kwanchai Jieam, Khammanithip Vongxay, Ken Choviran, Andrea Sbrilli, Massimo Gobbi, Carmela Centeno

**Affiliations:** a NIDA Center for Research & Development of Disaster Prevention & Management, School of Social and Environmental Development, National Institute of Development Administration (NIDA), 118 Moo 3, Sereethai Road, Klong-Chan, Bangkapi, Bangkok, Thailand; b Hazardous Substance Division, Waste and Hazardous Substance Management Bureau, Pollution Control Department (PCD), 92 Soi Phahonyothin 7, Phahonyothin Rd., Sam Sen Nai, Phayathai, Bangkok, Thailand; c Division of ASEAN Cooperation on Environment, Lao National Mekong Committee Secretariat, Ministry of Natural Resources and Environment, Lao Democratic People’s Republic; d Oleen Co., Ltd., Factory 64 Moo 6 Rama II Rd., Bangtorad, Muang, Samutsakhon, Thailand; e Lao Agro Industry Co., Ltd., Ban Kern, M. Thourakhom, Vientiane Province, Lao Democratic People’s Republic; f Deputy Director, Department of Pollution Control Ministry of Environment, 48, Samdech Preah Sihanouk, Tonle Bassac, Chamkarmon, Phnom Penh, Cambodia; g Industrial Development Officer, Stockholm Convention Unit, Environmental Management Branch, Wagramerstrasse 5, A-1400 Vienna, Austria

**Keywords:** Polychlorinated-dibenzo-para-dioxins (PCDDs), Polychlorinated-dibenzofurans (PCDFs), Best Available Technique (BAT), Best Environmental Practise (BEP), Industrial Boilers

## Abstract

Polychlorinated-dibenzo-para-dioxins (PCDDs) and Polychlorinated-dibenzofurans (PCDFs) contamination in ecosystems has been a major concern, however, no information is available about the atmospheric contents and emission profiles in different types of fuels from industrial boilers in Thailand, Lao PDR, and Cambodia. Nine air and bottom ash samples (*n* = 18) were collected from three industrial boilers using U.S.EPA Method 23 and U.S.EPA Method 8290, respectively. All samples were successfully quantified by two High Resolution Chromatography-High Resolution Mass Spectrometry (HRGC-HRMS) namely Waters Autospec Premier and Waters Autospec Ultima. This investigation elucidates the impacts of fuel type on the emissions of PCDDs and PCDFs from three different industrial boilers. It appears plausible to affirm that fuel types play an important role on PCDD/PCDF emission levels from industrial boilers. The results of PCDD/PCDF concentrations should be considered as baseline data for promoting Best Available Technique (BAT) and Best Environmental Practise (BEP) in order to reduce dioxin emissions from industrial boilers in Southeast Asian countries.

**Specifications table**

**Table:** 

Subject area	Environmental Sciences
More specific subject area	Environmental Chemistry
Type of data	Table, text file, graph, figure
How data was acquired	Soxhlet extraction and High Resolution Chromatography-High Resolution Mass Spectrometry (HRGC-HRMS) (Waters Autospec Premier and Waters Autospec Ultima) [1]
Data format	Raw data, analysed.
Experimental factors	PCDD/PCDF congeners were collected by using probe and hotbox system, console and sampling line, glassware kit, and Horizontal Modified Accessories Kit (APEX). Extraction is conducted in Soxhlet equipment with the appropriate solvent [2]. The clean-up procedure could be different from sample to sample, but a schematic of a typical process is given in Table 1.
Experimental features	PCDD/PCDF congeners using HRGC-HRMS.
Data source locations	All samples were collected from industrial boilers located at the Great Honour Textile Factory Ltd. (GHT), OLEEN Company Limited (Oleen), and Lai Agro Industry Company Limited (LAI). GHT is an exporter from Cambodia, the company sell ladies and gentlemen knitted pullover. Oleen is The largest and modernest edible palm oil producer in Thailand with 100% Thai Investment of 850 Millions Baht. LAI is one of the leading canned sweet corn production company in Lao PDR.
Data accessibility	Data available within article.

**Value of the data**

• Data can be employed as a base-line data for PCDD/PCDF concentration levels emitted from industrial boilers.
• There is always the possibility of environmental contamination with toxic substances such as PCDD/PCDF congeners, therefore, continuous air monitoring is obviously crucial for occupational health workers.
• Data displayed here may serve as benchmarks for other research groups focusing in the field of occupational safety and health, and toxicology to assess PCDD/PCDF congeners daily intakes by industrial emissions.
• The results of this dataset can be useful for United Nations Industrial Development Organization (UNIDO) to showcase the success of “Demonstration of BAT and BEP in Fossil Fuel-fired Utility and Industrial Boilers in Response to the Stockholm Convention on Persistent Organic Pollutants (POPs)”.

## 1 Data

Table 1, Table 2, Table 3 show descriptive statistics of selected PCDD/PCDF congeners collected from Oleen, LAI, and GHT, respectively. Table 4, Table 5, Table 6 are demonstrating the bottom ash concentration of PCDD/PCDF congeners collected at Oleen, LAI, and GHT, respectively.

**Table 1:** Atmospheric concentrations of PCDD/PCDF congeners (ng/Nm^3^) collected from Oleen.

**Parameters**	**TEF**	**Sampling date**									
		**10/06/15**				**11/06/15**			**12/06/15**		
		**ng/Nm**^**3**^	**at actual O**_**2**_	**at 20 °C and 7% O**_**2**_		**ng/Nm**^**3**^	**at actual O**_**2**_	**at 20 °C and 7% O**_**2**_	**ng/Nm**^**3**^	**at actual O**_**2**_	**at 20 °C and 7% O**_**2**_
			**(ng I-TEQ/Sm**^**3**^**)**				**(ng I-TEQ/Sm**^**3**^**)**			**(ng I-TEQ/Sm**^**3**^**)**	
**Determination of PCDD/PCDFs**											
**1.3 Total I-TEQ**											
1) 2,3,7,8-TCDD	1	0.0020	0.0020		0.0021	0.0011	0.0011	0.0012	0.0019	0.0019	0.0021
2) 1,2,3,7,8-PeCDD	0.5	0.0043	0.0021		0.0022	0.0013	0.0007	0.0007	< 0.0038	< 0.0019	< 0.0021
3) 1,2,3,4,7,8-HxCDD	0.1	0.0135	0.0014		0.0014	0.0012	0.0001	0.0001	0.0154	0.0015	0.0017
4) 1,2,3,6,7,8-HxCDD	0.1	0.0061	0.0006		0.0006	0.0011	0.0001	0.0001	0.0099	0.0010	0.0011
5) 1,2,3,7,8,9-HxCDD	0.1	0.0312	0.0031		0.0033	0.0008	0.0001	0.0001	0.0370	0.0037	0.0041
6) 1,2,3,4,6,7,8-HpCDD	0.01	< 0.0114	< 0.0001		< 0.0001	0.0058	0.0001	0.0001	< 0.0190	< 0.0002	< 0.0002
7) OCDD	0.001	< 0.0593	< 0.0001		< 0.0001	< 0.0116	< 0.00001	< 0.00001	< 0.0903	< 0.0001	< 0.0001
8) 2,3,7,8-TCDF	0.1	0.0059	0.0006		0.0006	< 0.0038	< 0.0004	< 0.0004	< 0.0038	< 0.0004	< 0.0004
9) 1,2,3,7,8-PeCDF	0.05	0.0138	0.0007		0.0007	0.0019	0.0001	0.0001	0.0071	0.0004	0.0004
10) 2,3,4,7,8-PeCDF	0.5	0.0378	0.0189		0.0198	0.0044	0.0002	0.0024	0.0193	0.0096	0.0107
11) 1,2,3,4,7,8-HxCDF	0.1	0.0179	0.0018		0.0019	0.0034	0.0003	0.0004	0.0147	0.0015	0.0016
12) 1,2,3,6,7,8-HxCDF	0.1	0.0125	0.0012		0.0013	0.0038	0.0004	0.0004	0.0108	0.0011	0.0012
13) 2,3,4,6,7,8-HxCDF	0.1	0.0050	0.0005		0.0005	0.0011	0.0001	0.0001	< 0.0047	< 0.0005	< 0.0005
14) 1,2,3,7,8,9-HxCDF	0.1	0.0052	0.0005		0.0005	0.0008	0.0001	0.0001	< 0.0047	< 0.0005	< 0.0005
15) 1,2,3,4,6,7,8-HpCDF	0.01	0.0281	0.0003		0.0003	0.0170	0.0002	0.0002	< 0.0190	< 0.0002	< 0.0002
16) 1,2,3,4,7,8,9-HpCDF	0.01	< 0.0114	< 0.0001		< 0.0001	0.0030	0.00003	0.00003	< 0.0190	< 0.0002	< 0.0002
17) OCDF	0.001	< 0.0593	< 0.0001		< 0.0001	0.0179	0.00002	0.00002	< 0.0903	< 0.0001	< 0.0001
**PCDD/PCDFs-TEQ**			**0.0337**		**0.0354**		**0.0055**	**0.0061**		**0.0207**	**0.0230**

Remarks:Sm^3^ =Dry standard cubic meter for gas condition means at temperature of 20 °C, pressure of 1 atm and dry basis. (United States Region)

I-TEQ (International Toxicity Equivalence) = the value is calculated by using the toxicity equivalence factors (TEF).

**Table 2:** Atmospheric concentrations of PCDD/PCDF congeners (ng/Nm^3^) collected from LAI.

**Parameters**	**TEF**	**Sampling date**									
		**26/06/12**				**27/06/12**			**28/06/12**		
		**ng/Nm**^**3**^	**at actual O**_**2**_	**at 20 °C and 7% O**_**2**_		**ng/Nm**^**3**^	**at actual O**_**2**_	**at 20 °C and 7% O**_**2**_	**ng/Nm**^**3**^	**at actual O**_**2**_	**at 20 °C and 7% O**_**2**_
			**(ng I-TEQ/Sm**^**3**^**)**				**(ng I-TEQ/Sm**^**3**^**)**			**(ng I-TEQ/Sm**^**3**^**)**	
**Determination of PCDD/PCDFs**											
**1.3 Total I-TEQ**											
18) 2,3,7,8-TCDD	1	< 0.0004	< 0.0004		< 0.0004	< 0.0004	< 0.0004	< 0.0004	< 0.0003	< 0.0003	< 0.0004
19) 1,2,3,7,8-PeCDD	0.5	0.0011	0.0006		0.0006	< 0.0007	< 0.0004	< 0.0004	< 0.0007	< 0.0003	< 0.0004
20) 1,2,3,4,7,8-HxCDD	0.1	0.0019	0.0002		0.0002	< 0.0009	< 0.0001	< 0.0001	< 0.0009	< 0.0001	< 0.0001
21) 1,2,3,6,7,8-HxCDD	0.1	0.0029	0.0003		0.0003	< 0.0009	< 0.0001	< 0.0001	< 0.0009	< 0.0001	< 0.0001
22) 1,2,3,7,8,9-HxCDD	0.1	0.0031	0.0003		0.0003	< 0.0009	< 0.0001	< 0.0001	< 0.0009	< 0.0001	< 0.0001
23) 1,2,3,4,6,7,8-HpCDD	0.01	0.0250	0.0003		0.0003	0.0088	0.0001	0.0001	0.0038	0.00004	0.00004
24) OCDD	0.001	0.0309	0.00003		0.00003	< 0.0187	< 0.00002	< 0.00002	< 0.0182	< 0.00002	< 0.00002
25) 2,3,7,8-TCDF	0.1	0.0016	0.0002		0.0002	0.0011	0.0001	0.0001	0.0009	0.0001	0.0001
26) 1,2,3,7,8-PeCDF	0.05	0.0018	0.0001		0.0001	0.0009	0.00005	0.00005	< 0.0007	< 0.00003	< 0.00004
27) 2,3,4,7,8-PeCDF	0.5	0.0035	0.0017		0.0018	0.0006	0.0003	0.0003	0.00003	0.00001	0.00002
28) 1,2,3,4,7,8-HxCDF	0.1	0.0027	0.0003		0.0003	0.0013	0.0001	0.0001	< 0.0009	< 0.0001	< 0.0001
29) 1,2,3,6,7,8-HxCDF	0.1	0.0025	0.0003		0.0003	0.0010	0.0001	0.0001	< 0.0009	< 0.0001	< 0.0001
30) 2,3,4,6,7,8-HxCDF	0.1	0.0037	0.0004		0.0004	0.0016	0.0002	0.0002	< 0.0009	< 0.0001	< 0.0001
31) 1,2,3,7,8,9-HxCDF	0.1	< 0.0009	< 0.0001		< 0.0001	< 0.0009	< 0.0001	< 0.0001	< 0.0009	< 0.0001	< 0.0001
32) 1,2,3,4,6,7,8-HpCDF	0.01	0.0063	0.0001		0.0001	0.0004	0.000004	0.000005	< 0.0035	< 0.00003	< 0.00004
33) 1,2,3,4,7,8,9-HpCDF	0.01	< 0.0037	< 0.00004		< 0.00004	< 0.0036	< 0.00004	< 0.00004	< 0.0035	< 0.00003	< 0.00004
34) OCDF	0.001	0.0236	0.00002		0.00002	< 0.0187	< 0.00002	< 0.00002	< 0.0182	< 0.00002	< 0.00002
**PCDD/PCDFs-TEQ**			**0.0046**		**0.0048**		**0.0010**	**0.0010**		**0.0001**	**0.0002**

Remarks:Sm^3^ = Dry standard cubic meter for gas condition means at temperature of 20 °C, pressure of 1 atm and dry basis. (United States Region)

ITEQ (International Toxicity Equivalence) = the value is calculated by using the toxicity equivalence factors (TEF).

**Table 3:** Atmospheric concentrations of PCDD/PCDF congeners (ng/Nm^3^) collected from GHT.

**Parameters**	**TEF**	**Sampling date**									
		**22/08/12**				**23/08/12**			**24/08/12**		
		**ng/Nm**^**3**^	**at actual O**_**2**_	**at 20 °C and 7% O**_**2**_		**ng/Nm**^**3**^	**at actual O**_**2**_	**at 20 °C and 7% O**_**2**_	**ng/Nm**^**3**^	**at actual O**_**2**_	**at 20 °C and 7% O**_**2**_
			**(ng I-TEQ/Sm**^**3**^**)**				**(ng I-TEQ/Sm**^**3**^**)**			**(ng I-TEQ/Sm**^**3**^**)**	
**Determination of PCDD/PCDFs**											
**1.3 Total I-TEQ**											
35) 2,3,7,8-TCDD	1	0.0388	0.0388		0.1033	0.0114	0.0114	0.0561	0.0163	0.0163	0.0485
36) 1,2,3,7,8-PeCDD	0.5	0.0707	0.0353		0.0941	0.0162	0.0081	0.0400	0.0278	0.0139	0.0412
37) 1,2,3,4,7,8-HxCDD	0.1	0.0305	0.0030		0.0081	0.0065	0.0006	0.0032	0.0129	0.0013	0.0038
38) 1,2,3,6,7,8-HxCDD	0.1	0.0513	0.0051		0.0137	0.0140	0.0014	0.0069	0.0245	0.0024	0.0073
39) 1,2,3,7,8,9-HxCDD	0.1	0.0443	0.0044		0.0118	0.0112	0.0011	0.0055	0.0196	0.0020	0.0058
40) 1,2,3,4,6,7,8-HpCDD	0.01	0.1537	0.0015		0.0041	0.0258	0.0003	0.0013	0.0586	0.0006	0.0017
41) OCDD	0.001	0.0443	0.00004		0.0001	< 0.0211	< 0.00002	< 0.0001	< 0.0212	< 0.00002	< 0.0001
42) 2,3,7,8-TCDF	0.1	0.3741	0.0374		0.0996	0.0959	0.0096	0.0473	0.1339	0.0134	0.0398
43) 1,2,3,7,8-PeCDF	0.05	0.1801	0.0090		0.0240	0.0422	0.0021	0.0104	0.0702	0.0035	0.0104
44) 2,3,4,7,8-PeCDF	0.5	0.3049	0.1524		0.4059	0.0747	0.0374	0.1842	0.1225	0.0612	0.1819
45) 1,2,3,4,7,8-HxCDF	0.1	0.1154	0.0115		0.0307	0.0233	0.0023	0.0115	0.0413	0.0041	0.0123
46) 1,2,3,6,7,8-HxCDF	0.1	0.1122	0.0112		0.0299	0.0227	0.0023	0.0112	0.0425	0.0042	0.0126
47) 2,3,4,6,7,8-HxCDF	0.1	0.1372	0.0137		0.0365	0.0277	0.0028	0.0136	0.0539	0.0054	0.0160
48) 1,2,3,7,8,9-HxCDF	0.1	0.0236	0.0024		0.0063	0.0026	0.0003	0.0013	0.0212	0.0021	0.0063
49) 1,2,3,4,6,7,8-HpCDF	0.01	0.1463	0.0015		0.0039	0.0221	0.0002	0.0011	0.0434	0.0004	0.0013
50) 1,2,3,4,7,8,9-HpCDF	0.01	0.0333	0.0003		0.0009	0.0063	0.0001	0.0003	0.0113	0.0001	0.0003
51) OCDF	0.001	0.0430	0.00004		0.0001	< 0.0211	< 0.00002	< 0.0001	< 0.0212	< 0.00002	< 0.0001
**PCDD/PCDFs-TEQ**			**0.3279**		**0.8730**		**0.0799**	**0.3938**		**0.1311**	**0.3893**

Remarks:Sm^3^ = Dry standard cubic meter for gas condition means at temperature of 20 °C, pressure of 1 atm and dry basis. (United States Region)

I-TEQ (International Toxicity Equivalence) = the value is calculated by using the toxicity equivalence factors (TEF).

**Table 4:** Bottom ash concentrations of PCDD/PCDF congeners (ng/kg) collected from Oleen.

**Parameters**	**TEF**	**Sampling date**					
		**10/06/15**		**11/06/15**		**12/06/15**	
		**Quantity (ng/kg)**	**Quantity (ng I-TEQ/kg)**	**Quantity (ng/kg)**	**Quantity (ng I-TEQ/kg)**	**Quantity (ng/kg)**	**Quantity (ng I-TEQ/kg)**
**Sampling Time**	–	13.30,16.30,19.30		14.00,17.00,20.00		14.00,17.00,20.00	
1. **Total PCDD**^a^							
1) TCDDs	–	86	–	440	–	36	–
2) PeCDDs	–	42	–	140	–	12	–
3) HxCDDs	–	18	–	41	–	11	–
4) HpCDDs	–	4.2	–	4.5	–	2.3	–
2. **Total PCDF**^b^							
1) TCDFs	–	1.6	–	14	–	< 1.0	–
2) PeCDFs	–	< 0.90	–	2.0	–	1.5	–
3) HxCDFs	–	1.1	–	3.5	–	11	–
4) HpCDFs	–	1.8	–	3.1	–	2.3	–
3. **Total I-TEQ.**^c^							
1) 2,3,7,8-TCDD	1	< 0.90	< 0.90	< 0.75	< 0.75	< 1.0	< 1.0
2) 1,2,3,7,8-PeCDD	0.5	< 0.90	< 0.45	< 0.75	< 0.37	< 1.0	< 0.52
3) 1,2,3,4,7,8-HxCDD	0.1	< 0.90	< 0.090	0.88	0.088	< 1.0	< 0.10
4) 1,2,3,6,7,8-HxCDD	0.1	< 0.90	< 0.090	< 0.75	< 0.075	< 1.0	< 0.10
5) 1,2,3,7,8,9-HxCDD	0.1	< 0.90	< 0.090	< 0.75	< 0.075	< 1.0	< 0.10
6) 1,2,3,4,6,7,8-HpCDD	0.01	2.2	0.022	3.0	0.030	< 1.7	< 0.017
7) OCDD	0.001	4.2	0.0042	3.5	0.0035	< 3.5	< 0.0035
8) 2,3,7,8-TCDF	0.1	< 0.9	< 0.090	< 0.75	< 0.075	< 1.0	< 0.10
9) 1,2,3,7,8-PeCDF	0.05	< 0.90	< 0.045	< 0.75	< 0.037	1.0	0.052
10) 2,3,4,7,8-PeCDF	0.5	< 0.90	< 0.45	< 0.75	< 0.37	< 1.0	< 0.52
11) 1,2,3,4,7,8-HxCDF	0.1	< 0.90	< 0.090	0.75	0.075	< 1.0	< 0.10
12) 1,2,3,6,7,8-HxCDF	0.1	< 0.90	< 0.090	< 0.75	< 0.075	< 1.0	< 0.10
13) 2,3,4,6,7,8-HxCDF	0.1	< 0.90	< 0.090	< 0.75	< 0.075	< 1.0	< 0.10
14) 1,2,3,7,8,9-HxCDF	0.1	< 0.90	< 0.090	< 0.75	< 0.075	< 1.0	< 0.10
15) 1,2,3,4,6,7,8-HpCDF	0.01	< 1.5	< 0.015	< 1.9	< 0.019	< 1.7	< 0.017
16) 1,2,3,4,7,8,9-HpCDF	0.01	< 1.5	< 0.015	< 1.2	< 0.012	< 1.7	< 0.017
17) OCDF	0.001	< 3.0	< 0.0030	< 2.5	< 0.0025	< 3.5	< 0.0035

a Polychlorinated Dibenzo-p-Dioxins.

b Polychlorinated Dibenzofurans.

c I-TEQ (International Toxicity Equivalence) = the value is calculated by using the toxicity equivalence factors (TEF).

**Table 5:** Bottom ash concentrations of PCDD/PCDF congeners (ng/kg) collected from LAI.

**Parameters**	**Quantity (ng/kg)**	**TEF**	**Quantity (ng I-TEQ/kg)**
1. **Total PCDD**^a^			
1) TCDDs	0.79	–	–
2) PeCDDs	0.93	–	–
3) HxCDDs	1.6	–	–
4) HpCDDs	< 1.2	–	–
2. **Total PCDF**^b^			
1) TCDFs	1.5	–	–
2) PeCDFs	1.2	–	–
3) HxCDFs	2.1	–	–
4) HpCDFs	21	–	–
3. **Total I**–**TEQ**^c^			
1) 2,3,7,8-TCDD	< 0.73	1	< 0.73
2) 1,2,3,7,8-PeCDD	< 0.73	0.5	< 0.36
3) 1,2,3,4,7,8-HxCDD	< 0.73	0.1	< 0.073
4) 1,2,3,6,7,8-HxCDD	< 0.73	0.1	< 0.073
5) 1,2,3,7,8,9-HxCDD	< 0.73	0.1	< 0.073
6) 1,2,3,4,6,7,8-HpCDD	< 1.2	0.01	< 0.012
7) OCDD	3.9	0.001	0.0039
8) 2,3,7,8-TCDF	< 0.73	0.1	< 0.073
9) 1,2,3,7,8-PeCDF	< 0.73	0.05	< 0.036
10) 2,3,4,7,8-PeCDF	< 0.73	0.5	< 0.36
11) 1,2,3,4,7,8-HxCDF	< 0.73	0.1	< 0.073
12) 1,2,3,6,7,8-HxCDF	< 0.73	0.1	< 0.073
13) 2,3,4,6,7,8-HxCDF	< 0.73	0.1	< 0.073
14) 1,2,3,7,8,9-HxCDF	< 0.73	0.1	< 0.073
15) 1,2,3,4,6,7,8-HpCDF	14	0.01	0.14
16) 1,2,3,4,7,8,9-HpCDF	2.5	0.01	0.025
17) OCDF	440	0.001	0.44

a Polychlorinated Dibenzo-p-Dioxins.

b Polychlorinated Dibenzofurans.

c I-TEQ (International Toxicity Equivalence) = the value is calculated by using the toxicity equivalence factors (TEF).

**Table 6:** Bottom ash concentrations of PCDD/PCDF congeners (ng/kg) collected from GHT.

**Sampling Date**		**22/08/2012**		**23/08/2012**		**24/08/2012**	
**Sampling Time**		**14.00, 16.30, 18.00**		**10.30, 14.00, 16.00**		**10.30, 12.00, 14.30**	
**Parameters**	**TEF**	**Quantity (ng/kg)**	**Quantity (ng I-TEQ/kg)**	**Quantity (ng/kg)**	**Quantity (ng I-TEQ/kg)**	**Quantity (ng/kg)**	**Quantity (ng I-TEQ/kg)**
1. **Total PCDD**^a^							
1) TCDDs	–	31	–	< 0.75	–	0.97	–
2) PeCDDs	–	26	–	< 0.75	–	< 0.75	–
3) HxCDDs	–	23	–	< 0.75	–	< 0.75	–
4) HpCDDs	–	13	–	< 1.2	–	< 1.2	–
2. **Total PCDF**^b^							
1) TCDFs	–	130	–	5.8	–	7.5	–
2) PeCDFs	–	79	–	< 0.75	–	< 0.75	–
3) HxCDFs	–	53	–	1.1	–	< 0.75	–
4) HpCDFs	–	27	–	< 1.2	–	< 1.2	–
3. **Total I–TEQ**^c^							
1) 2,3,7,8-TCDD	1	0.82	0.82	< 0.75	< 0.75	< 0.75	< 0.75
2) 1,2,3,7,8-PeCDD	0.5	1.5	0.73	< 0.75	< 0.37	< 0.75	< 0.37
3) 1,2,3,4,7,8-HxCDD	0.1	0.98	0.098	< 0.75	< 0.075	< 0.75	< 0.075
4) 1,2,3,6,7,8-HxCDD	0.1	1.2	0.12	< 0.75	< 0.075	< 0.75	< 0.075
5) 1,2,3,7,8,9-HxCDD	0.1	1.5	0.15	< 0.75	< 0.075	< 0.75	< 0.075
6) 1,2,3,4,6,7,8-HpCDD	0.01	6.4	0.064	< 1.2	< 0.012	< 1.2	< 0.012
7) OCDD	0.001	11	0.011	< 2.5	< 0.0025	< 2.5	< 0.0025
8) 2,3,7,8-TCDF	0.1	6.2	0.62	< 0.75	< 0.075	1.1	0.11
9) 1,2,3,7,8-PeCDF	0.05	5.0	0.25	< 0.75	< 0.037	< 0.75	< 0.037
10) 2,3,4,7,8-PeCDF	0.5	5.6	2.8	< 0.75	< 0.37	< 0.75	< 0.37
11) 1,2,3,4,7,8-HxCDF	0.1	4.6	0.46	< 0.75	< 0.075	< 0.75	< 0.075
12) 1,2,3,6,7,8-HxCDF	0.1	5.0	0.50	< 0.75	< 0.075	< 0.75	< 0.075
13) 2,3,4,6,7,8-HxCDF	0.1	6.4	0.64	< 0.75	< 0.075	< 0.75	< 0.075
14) 1,2,3,7,8,9-HxCDF	0.1	< 0.75	< 0.075	< 0.75	< 0.075	< 0.75	< 0.075
15) 1,2,3,4,6,7,8-HpCDF	0.01	16	0.16	< 1.2	< 0.012	< 1.2	< 0.012
16) 1,2,3,4,7,8,9-HpCDF	0.01	2.6	0.026	< 1.2	< 0.012	< 1.2	< 0.012
17) OCDF	0.001	9.4	0.0094	< 2.5	< 0.0025	< 2.5	< 0.0025

a Polychlorinated Dibenzo-p-Dioxins.

b Polychlorinated Dibenzofurans.

c I-TEQ (International Toxicity Equivalence) = the value is calculated by using the toxicity equivalence factors (TEF).

## 2 Experimental design, materials and methods

### 2.1 Dataset area

All samples were collected from industrial boilers located at the Great Honour Textile Factory Ltd. (GHT), OLEEN Company Limited (Oleen), and Lai Agro Industry Company Limited (LAI) (see Table S1 and Fig. S2–S4 in Supporting material). All fuels used in three industrial boilers of GHT, OLEEN, and LAI were cashew wood, anthracite coal, and heavy oil grade C, respectively.

### 2.2 Sample collection and analytical procedures

Sampling and analytical methods of PCDD/PCDFs, Total Suspended Particulate (TSP), air temperature, air pressure, air velocity, flow rate, moisture content, oxygen (O_2_), carbon monoxide (CO), carbon dioxide (CO_2_), sulphur dioxide (SO_2_), oxide of nitrogen (NO_x_ as NO_2_) and total chloride are measured in 2015 and described in Table S2. All details of equipment for emission air measurement such as probe and hotbox system, console and sampling line, Method 5 glassware kit, and Method 23 Horizontal Modified Accessories Kit (APEX) are clearly explained in Tables S3 [3], [4]. The information of traverse points number coupled with fraction of stacks diameter from inside wall to traverse point are fully described in Table S4. In addition, all details of methodologies (i.e. Method 1–4) for emission air measurement are comprehensively written in Tables S5 [5], [6]. All chemical analyses of the PCDD/PCDFs were conducted at SGS environmental laboratories that have ISO 17025 accreditations and demonstrate the effectiveness to properly execute testing methods practices, inspection routines, data validation and employee competences. ISO 17025 is an international standard, which sets the general requirements for the competence of testing and calibration laboratories. In this study, 17 congeners of PCDD/PCDFs were qualitatively and quantitatively analyzed to include the following: 2,3,7,8-TCDD, 1,2,3,7,8-PeCDD, 1,2,3,4,7,8-HxCDD, 1,2,3,6,7,8-HxCDD, 1,2,3,7,8,9-HxCDD, 1,2,3,4,6,7,8-HpCDD, OCDD, 2,3,7,8-TCDF, 1,2,3,7,8-PeCDF, 2,3,4,7,8-PeCDF, 1,2,3,4,7,8-HxCDF, 1,2,3,6,7,8-HxCDF, 2,3,4,6,7,8-HxCDF, 1,2,3,7,8,9-HxCDF, 1,2,3,4,6,7,8-HpCDF, 1,2,3,4,7,8,9-HpCDF, and OCDF. A clear description for dioxin analysis is given in Fig. S1 as a flowchart. In addition, the QA/QC requirements for exhaust air measurements were as follows: (*i*) the isokinetic rate must be examined, and the result must be during 90–110%; (*ii*) the sampling equipment must be checked for leaks and proper calibration, (*iii*) the sample control using chain of custody (COC) form must be used for sample delivery and control to and within the laboratory; (*iv*) field blank to checks to determine any contamination must be conducted. In addition, the QA/QC examinations of the analyses were conducted by calculating blanks and recoveries as clearly described in Table S1B. The average recovery rate of ^13^C labeled 2,3,7,8-chlorine substituted dioxins was 81.7±9.38%. The precisions and accuracies were assured by using NIST (National Institute of Standards and Technology)-SRM (Standard Reference Material)-1649b urban dust. In addition, two HRGC-HRMS namely Waters Autospec Premier and Waters Autospec Ultima were used in this study. The QA & QC data for recovery efficiency of PCDD/PCDFs analysis were clearly displayed in Table S6.
