# Supplementary material for: Data relating to emissions of polychlorinated dibenzo-p-dioxins (PCDDs) and polychlorinated dibenzofurans (PCDFs) from industrial boilers
Source: Data Brief. 2018 Dec 13;22:286–95. doi: 10.1016/j.dib.2018.12.021 (PMC6305761; doi:10.1016/j.dib.2018.12.021)
Supplement: Supplementary file 2 — Supplementary material [file mmc2.docx]

**Table S1.** Descriptions of sampling sites and industrial boiler types.

| **Name** | **Address and Boiler Types** |
| --- | --- |
| GHT | GHT is an exporter from Cambodia, the company sell ladies and gentlemen knitted pullover and situated at Villageno 4 Nat L Rd No 21, Commune Svay Rolum, District Sa Ang, Province Kandal, Cambodia. |
| Oleen | Oleen is The largest and modernest edible palm oil producer in Thailand with 100% Thai Investment of 850 Millions Baht. Oleen is located at 64 Moo 6, Rama II Rd, Muang, Samutsakorn, Thailand. |
| LAI | LAI is one of the leading canned sweet corn production company in Lao PDR. LAI is situated at Ban Kern M. Thourakhom, Vientiane Province, Lao PDR. |

**Table S2.** Sampling and analytical methods.

| **Environmental Quality/Parameter** | **Sampling/Analysis Method** |
| --- | --- |
| 1. Emission Air Monitoring   • PCDD/PCDFs  • Total Suspended Particulate (TSP)  • Temperature  • Pressure   • Velocity  • Flow rate  • Moisture Content  • Oxygen (O_2_)  • Carbon Monoxide (CO)  • Carbon Dioxide (CO_2_)  • Sulfur Dioxide (SO_x_)  • Oxide of Nitrogen (NO_x_ as NO_2_) | • U.S.EPA Method 23 / HRGC-HRMS  • U.S.EPA Method 5 / Gravimetric Method  • U.S.EPA Method 2 / S-Type Pitot Tube   • U.S.EPA Method 2 / S-Type Pitot Tube  • U.S.EPA Method 2 / S-Type Pitot Tube   • U.S.EPA Method 2 / S-Type Pitot Tube   • U.S.EPA Method 4 / Condensate Technique  • Portable Detector (Testo)  • Portable Detector (Testo)  • Portable Detector (Testo)  • Portable Detector (Testo)  • Portable Detector (Testo) |
| 2. Solid Residue  • PCDD/PCDFs | • U.S.EPA Method 8290 and 1613 / HRGC-HRMS |
| 3. Fuel Oil  • Total Chloride^a^ | • ASTM D 5808 |

Remark: _a_Fuel oil will be analyzed for total chloride instead of total chlorine.

**Table S3.** List of equipment for emission air measurement.

| **Probe and Hot box System** |
| --- |
| Heated Filter Box with 2 Access Doors , Probe Clamp and impinger box slide,220 V |
| Compact Method 5 82mm SS Heated Fitter Assembly |
| 5 Foot Glass Lined Method 5 Probe Assembly, 220 V |
| NOZZLE, SET OF Glass Nozzles-Sizes 4,6,8,10,12,14 and 16,includind Case and 3 5/8 inch Tube Nuts 4 inch Filter Assembly, Unground Glass, Teflon Filter Support, OZ Style Threaded Filter Clamp Double L Adapter,Unground # 28 Socket |
| **Console and Sampling Line** |
| Metering Console Model XC-572 with Metric Numeric Display Gas Meter ,Dual Column Inclined Manometer ,Digital Temperature Display ,Solid state Timer ,Solid State Temperature Controllers and External Rotary Vane Pump Assembly , 220 VAC. |
| External Pump Assembly ,Vane pump,Lubricator,SS Quick Connect,5ft Pwr Cord & houses,240 V Compact Method 5 Umbilical Cord , 90 foot sample Line and Exit TC cut-back, 15 feet brass fittings Sample case/ Umbilical Adapter |
| Nozzle Brush Set (Size 3,5,&8 ) in Carrying Tube |
| Modular Probe Brush Extension Kit , Nylon Brushes with Stainless Steel Extension |
| **Method 5 Glassware Kit** |
| Impinger Assembly, Plain Stem,500 ml., Unground O-ring Joints, Modified Greenburg-Smith Impinger Assembly, Stem with Orifice & Plate, 500 ml., Unground O-ring Joints, Greenburg-Smith U-Tube #28, 28Un ground Sockets |
| #28 SS Ball Joint Clamp |
| **Method 23 Horizontal Modified Accessories Kit (APEX)** |
| Impinger Box, Model 150 |
| Horizontal Condenser, Unground |
| Knock out Impinger, Unground |
| Submersible Coolant Pump, 220V |
| XAD Trap, #28 Ball & Socket Joints, Unground Glass Cap #28 Socket, Unground, to Seal XAD Trap Glass Plug #28 Ball, Unground , to Seal XAD Trap Keck Clip #29 Plastic Ball Joint Clamp |
| XAD Thermocouple Assembly |
| Ft of Latex Tubing, 7/16 OD, 5/16 ID, Natural Color Case, Transport, Glass Ware (Local Supply) |

**Table S4.** Location of traverse point in circular stacks.

| Traverse Points Number | Fraction of Stacks Diameter from Inside Wall to Traverse Point | | | | | |
| --- | --- | --- | --- | --- | --- | --- |
|  | Number of Traverse Point on a Diameter | | | | | |
|  | 2 | 4 | 6 | 8 | 10 | 12 |
| 1 | 0.146 | 0.674 | 0.044 | 0.032 | 0.026 | 0.021 |
| 2 | 0.854 | 0.25 | 0.146 | 0.105 | 0.082 | 0.067 |
| 3 |  | 0.75 | 0.296 | 0.194 | 0.146 | 0.118 |
| 4 |  | 0.933 | 0.704 | 0.323 | 0.226 | 0.177 |
| 5 |  |  | 0.854 | 0.677 | 0.342 | 0.25 |
| 6 |  |  | 0.956 | 0.806 | 0.658 | 0.356 |
| 7 |  |  |  | 0.895 | 0.774 | 0.644 |
| 8 |  |  |  | 0.968 | 0.854 | 0.75 |
| 9 |  |  |  |  | 0.918 | 0.823 |
| 10 |  |  |  |  | 0.974 | 0.882 |
| 11 |  |  |  |  |  | 0.933 |
| 12 |  |  |  |  |  | 0.97 |

**Table S5.** Methodology for emission air measurement.

| **Method 1** – Selection of Sampling Site and Determining Sample and Velocity Traverse Point |
| --- |
| This Method describes procedure to select an appropriate sampling location on the stacks.  * For circular stack with diameters greater than 60 cm, the minimum number of traverse point required is twelve, or six in each of two or more downstream.  * For rectangular the minimum number of traverse points required is nine or 3×3. Calculate the location of the traverse point for velocity and particulate sampling within the stack. |
| **Method 2** – Stack Gas Velocity and Volumetric Flow Rate. |
| Velocity measurement in a duct is made using a pitot tube type-S that is connected to an inclined manometer. The S-Type pitot tube is into the stack, so that one leg (hole opening) of the pitot tube is pointing into the direction of gas flow. The leg pointing into the flow streamline measures impact pressure P_i_ and the opposite leg pointing away from the flow measures wake pressure P_w_ of the gas steam. The velocity pressure Δp is the difference between the impact and wake pressure:  Δp=P_i_–P_w_  The procedure for determining flow rate in a stack gas steam is as follows:  1. Fill out the top section of a velocity Traverse field data sheet.  2. Have the point tube marked for traverse points according to Method 1.  3. Assemble the apparatus for flow velocity measurement:  a. Pitot tube with thermocouple, point and thermocouple extension lines, inclined manometer, temperature display device.  b. Use Probe Assembly, Umbilical Cable and inclined manometer on Meter Console.  4. Adjust Level and zero the manometer.  5. Insert the pitot tube in to the stack to a marked traverse point, seal off the port opening with a rag or towel to prevent ambient effects. Measure the velocity head and temperature, and record on the field data sheet.  6. Move to each traverse point, and record the velocity head and temperature.  7. Measure the static pressure in the stack.  8. Determine the barometric pressure at sample port level.  9. Calculate the average stack temperature from the traverse reading and record.  10. Calculate the average square root of velocity head by squaring each velocity head reading, summing the squares, and averaging (divided by number of traverse points), then record on the field data sheet. |
| **Method 3** – Gas Analysis for Dry Molecular Weight |
| Method 3 is used measure the percent concentrations of carbon dioxide (CO_2_), oxygen (O_2_), and carbon monoxide (CO) if greater than 0.2%. Nitrogen (N_2_) is calculated by difference. Form this data, the stack gas dry molecular weight, or density, is calculated, and this data is used in the equation for stack gas velocity. From the gas composition data, the amount of excess air for combustion sources can be calculated. In jurisdiction where the particulate emissions are regulated on concentration basis, such as mg m^-3^, the gas composition data can be used to correct the concentration result to a reference diluent concentration, for example 7% O_2_ or 12% CO_2_. |
| **Method 4** – Determination of Moisture Content |
| A gas sample is extracted at a constant rate from the source; moisture is removed from the sample stream and determined either volumetrically or gravimetrically. |

**Table S6.** The QA&QC data for recovery efficiency of PCDD/PCDFs analysis.

| **Component** | **Recovery Sampling Standards (%)** | | | | | | | |
| --- | --- | --- | --- | --- | --- | --- | --- | --- |
|  | **Blank** | **No.1** | **No.2** | **No.3** | **Blank** | **No.4** | **No.5** | **No.6** |
| ^13^C-2,3,4,7,8-PeCDF | 98.7 | 91.5 | 100 | 96.2 | 110 | 199 | 128 | 459 |
| ^13^C-1,2,3,4,7,8-HxCDF | 87.3 | 87.3 | 94.4 | 91.0 | 104 | 146 | 143 | 379 |
| ^13^C-1,2,3,4,7,8,9-HpCDF | 76.6 | 96.1 | 108 | 95.2 | 87.6 | 82.4 | 124 | 212 |
| ^13^C-2,3,7,8-TCDD | 94.9 | 86.2 | 90.8 | 85.3 | 104 | 103 | 135 | 250 |
| ^13^C-1,2,3,4,7,8-HxCDD | 84.5 | 85.9 | 96.4 | 90.6 | 96.2 | 125 | 121 | 519 |
| **Component** | **Recovery extraction Standards (%)** | | | | | | | |
|  | **Blank** | **No.1** | **No.2** | **No.3** | **Blank** | **No.4** | **No.5** | **No.6** |
| ^13^C-2,3,7,8-TCDF | 64.9 | 82.9 | 83.5 | 87.5 | 84.1 | 56.0 | 72.9 | 107 |
| ^13^C-1,2,3,7,8-PeCDF | 67.0 | 84.9 | 85.1 | 79.2 | 85.7 | 66.2 | 89.3 | 59.6 |
| ^13^C-1,2,3,6,7,8-HxCDF | 99.9 | 103 | 101 | 103 | 72.2 | 99.8 | 65.7 | 105 |
| ^13^C-1,2,3,4,6,7,8-HpCDF | 80.9 | 78.7 | 70.8 | 87.7 | 74.0 | 90.4 | 75.0 | 115 |
| ^13^C-2,3,7,8-TCDD | 71.4 | 94.9 | 93.1 | 101 | 89.2 | 14.2 | 71.6 | 18.1 |
| ^13^C-1,2,3,7,8-PeCDD | 70.1 | 92.5 | 95.7 | 81.5 | 110 | 132 | 103 | 94.9 |
| ^13^C-1,2,3,6,7,8-HxCDD | 96.3 | 99.9 | 102 | 105 | 75.6 | 19.6 | 76.2 | 18.1 |
| ^13^C-1,2,3,4,6,7,8-HpCDD | 78.4 | 89.9 | 93.1 | 95.8 | 85.6 | 114 | 91.0 | 87.1 |
| ^13^C-OCDD | 58.1 | 85.3 | 95.9 | 86.3 | 72.6 | 96.3 | 81.8 | 74.3 |


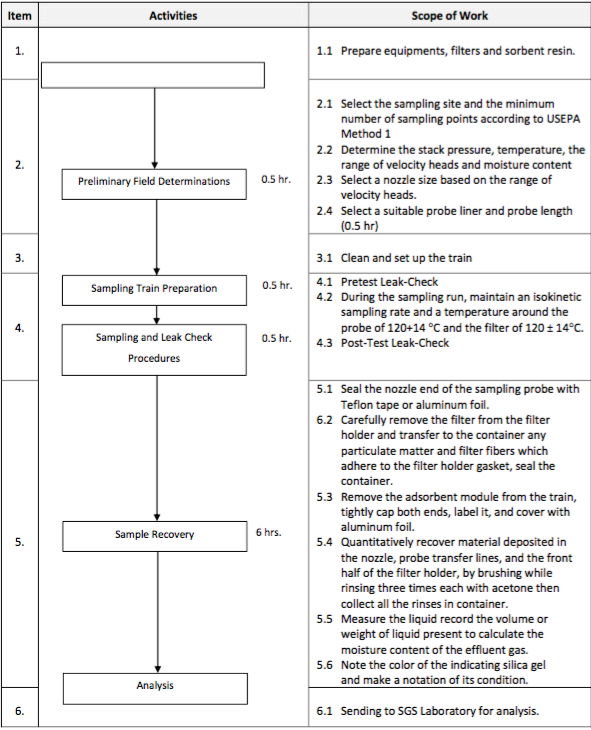


**Fig. S1.** Flowchart of sampling and analysis procedure for dioxin (US EPA Method 23) from stationary sources.

| 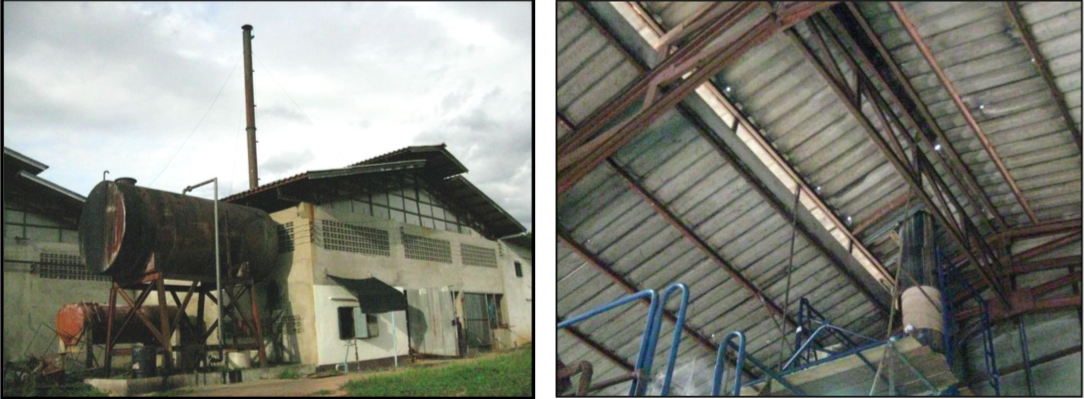 |
| --- |
| 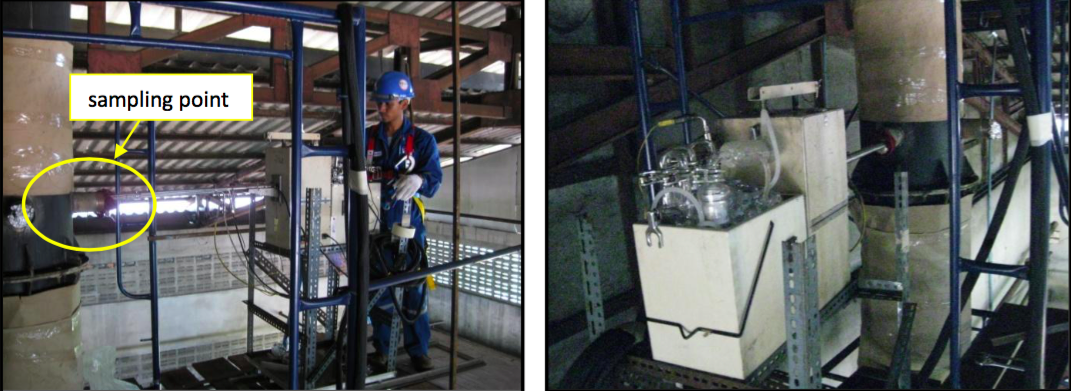 |
| 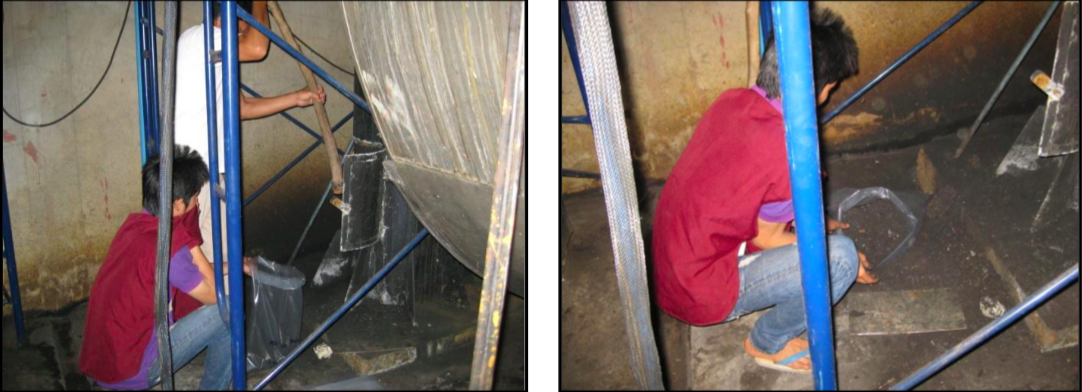 |

**Fig. S2.** Emission air and ash samplings with a boiler stack at LAI on June 28^th^, 2012.

| 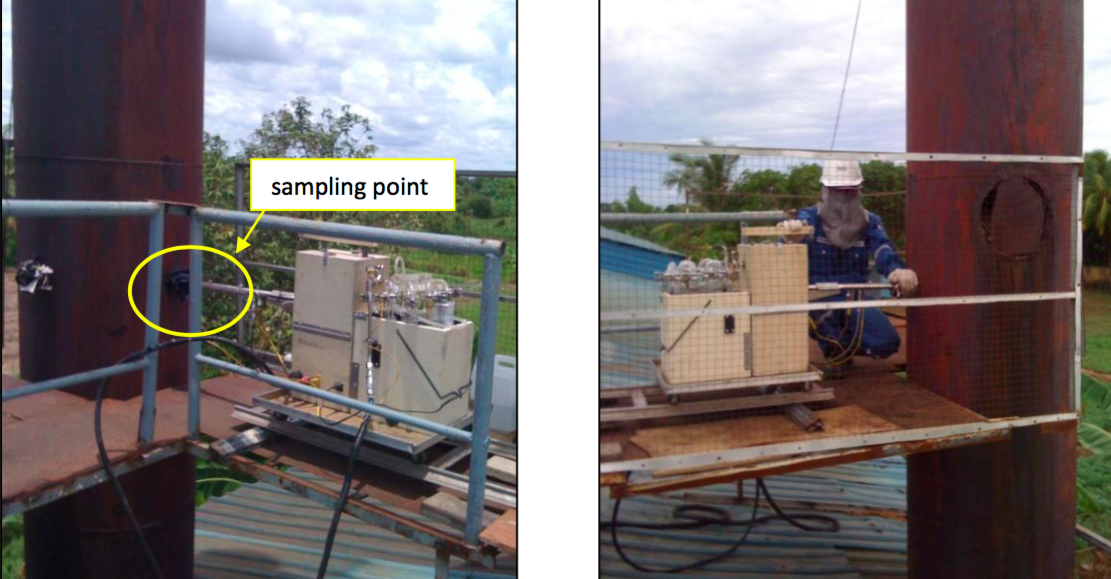 |
| --- |
| 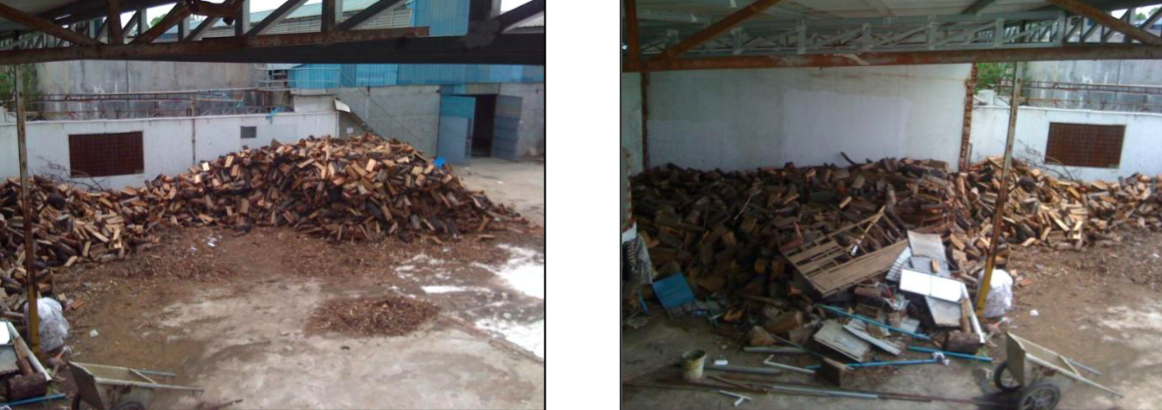 |
| 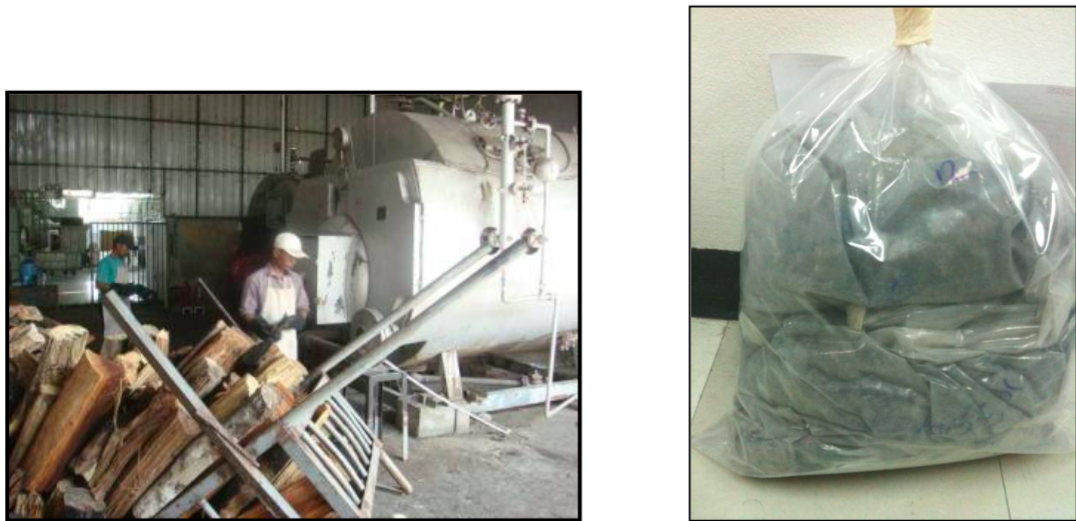 |

**Fig. S3.** Emission air and ash samplings with a boiler stack at GHT on August 22-25, 2012.

| **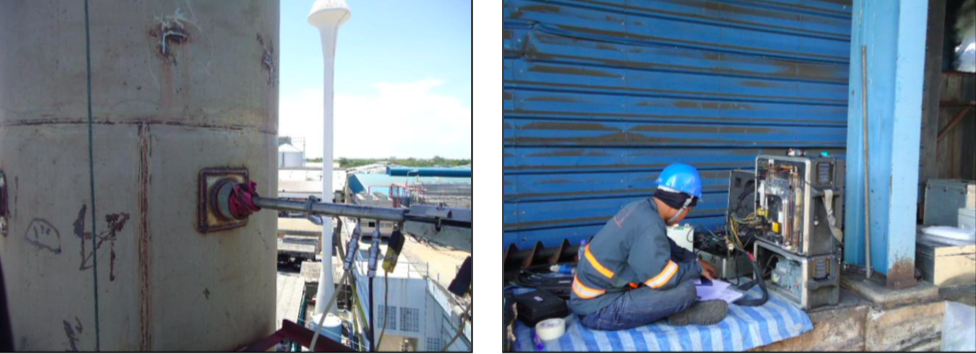** |
| --- |
| **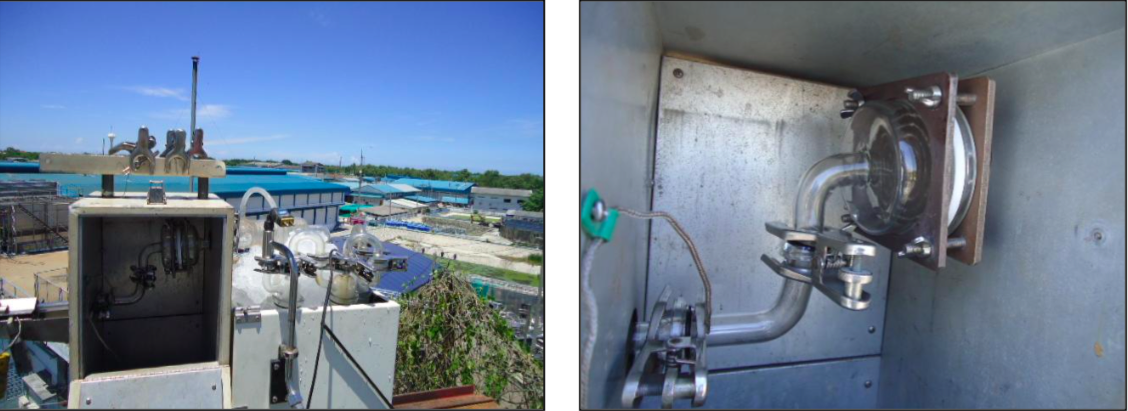** |
| **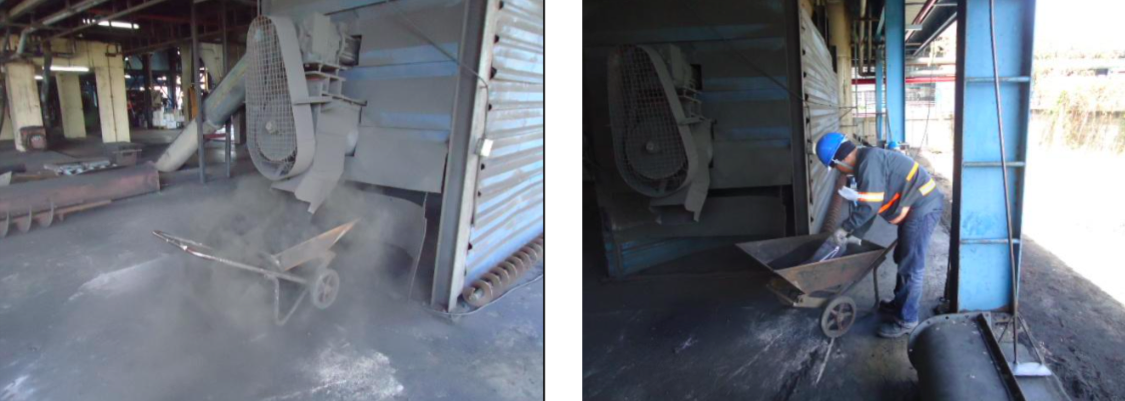** |

**Fig. S4.** Emission air and ash samplings with a boiler stack at Oleen on June 9-12, 2015.
