# Supplementary material for: Data relating to emissions of polychlorinated dibenzo-p-dioxins (PCDDs) and polychlorinated dibenzofurans (PCDFs) from industrial boilers
Source: Data Brief. 2018 Dec 13;22:286–95. doi: 10.1016/j.dib.2018.12.021 (PMC6305761; doi:10.1016/j.dib.2018.12.021)
Supplement: Supplementary file 1 — Transparency document [file mmc1.docx]

**Conflicts of Interest Statement**

04.12.18

Dear Editor of DIB,

**Manuscript title:** Data Relating to Emissions of Polychlorinated Dibenzo-p-Dioxins (PCDDs) and Polychlorinated Dibenzofurans (PCDFs) from Industrial Boilers

The corresponding author certifies that they have NO affiliations with or involvement in any organization or entity with any financial interest (such as honoraria; educational grants; participation in speakers’ bureaus; membership, employment, consultancies, stock ownership, or other equity interest; and expert testimony or patent-licensing arrangements), or non-financial interest (such as personal or professional relationships, affiliations, knowledge or beliefs) in the subject matter or materials discussed in this manuscript.

Best Regards,

Prof. Dr. Siwatt Pongpiachan

School of Social & Environmental Development

National Institute of Development Administration

118 Sereethai Road, Klongchan, Bangkapi, Bangkok

Thailand 10240
